# Decrease in an Inwardly Rectifying Potassium Conductance in Mouse Mammary Secretory Cells after Forced Weaning

**DOI:** 10.1371/journal.pone.0141131

**Published:** 2015-10-20

**Authors:** Akihiro Kamikawa, Shota Sugimoto, Osamu Ichii, Daisuke Kondoh

**Affiliations:** 1 Division of Biomedical Science, Department of Basic Veterinary Medicine, Obihiro University of Agriculture and Veterinary Medicine, Obihiro, Japan; 2 Laboratory of Anatomy, Department of Biomedical Sciences, Graduate School of Veterinary Medicine, Hokkaido University, Sapporo, Japan; University at Buffalo, UNITED STATES

## Abstract

Mammary glands are physiologically active in female mammals only during nursing. Immediately after weaning, most lactation-related genes are downregulated and milk production ceases. In our previous study, we have detected an inwardly rectifying potassium channel (Kir) 2.1-like current in mammary secretory (MS) cells freshly isolated from lactating mice. This current is highly sensitive to external Ba^2+^. The potassium permeability of the Kir channels may contribute to the secretion and/or preservation of ions in milk. We hypothesized that the functions of the Kir channels in MS cells are regulated after weaning. To test this hypothesis, we examined the effect of forced weaning on the Ba^2+^-sensitive Kir current and Kir2.1 expression in the mouse mammary glands. Twenty-four hours after weaning, the lumina of mammary acini were histologically enlarged by milk accumulation. The whole-cell patch-clamp analyses showed that the Ba^2+^-sensitive Kir current in the post-weaning MS cells was smaller than in the lactating MS cells. The inward conductances of the current in the lactating and post-weaning cells were 4.25 ± 0.77 and 0.93 ± 0.34 nS, respectively. Furthermore, real-time PCR and Western blot analyses showed that Kir2.1 mRNA and protein expression decreased in the post-weaning mammary gland (mRNA, 90% reduction; protein, 47% reduction). Moreover, the local milk accumulation caused by teat sealing decreased Kir conductance in MS cells (2.74 ± 0.45 and 0.36 ± 0.27 nS for control and sealed mammary glands, respectively). This was concomitant with the reduction in the Kir2.1 mRNA expression. Our results suggest that milk stasis after weaning immediately decreases the Kir conductance in MS cells. This decrease in the Kir conductance may be partly caused by the reduction in the Kir2.1 mRNA and protein expression. These alterations during the post-weaning period may be involved in the cessation of ion secretion and/or preservation in the milk.

## Introduction

The mammary gland is an exocrine gland that functions only during the lactation period in female mammals. Mammary acini develop on the mammary ducts during pregnancy, and acinar cells differentiate into secretory cells. After parturition, the milk produced by the secretory cells is stored in the lumina and ejected from the teats in response to suckling stimuli, providing nutrition to the pups. The mammary glands stop milk production and begin to involute within a few days after weaning [1]. The mechanisms by which lactation stops after weaning are not clear. To understand these mechanisms in detail, we focused on the alterations in ion permeabilities of MS cells after weaning.

Milk contains not only organic compounds but also mineral ions like K^+^, Na^+^, and Cl^−^. The vectorial ion transport by ion transporters and channels may provide the driving force for fluid secretion and contribute to the regulation of the volume and content of milk. This notion has been based on the data obtained for the ion concentrations in milk, blood, and secretory cells and transepithelial potential differences in mammary glands of ruminants and rodents [2, 3, 4]. In addition, the studies performed using monolayers of cultured mammary epithelial cells have revealed that the ions are selectively transported through K^+^, Na^+^, and Cl^−^ channels [5, 6, 7]. These findings have strongly suggested that, during lactation, the ion transport is an important function of MS cells. However, limited data is available regarding the functional ion channels in completely differentiated MS cells of lactating animals. In our previous study, we detected an inwardly rectifying K^+^ (Kir) current, at least partly carried by Kir2.1, in MS cells of lactating mice [8]. We have found that the Kir channels are constitutively active and their inhibition induces depolarization of zero-current voltage in a current-clamp analysis. Therefore, the Kir2.1-like channel may contribute to the maintenance of negative resting membrane potential, which could provide the driving force for ion movement and continuous secretion of the aqueous phase of milk [8].

Mammary gland involution has been examined in mice after forced weaning by removal of the pups. The lumen continues to enlarge during ~12 h after pup removal because of the milk accumulation. However, its size does not increase any further during the 12–24 h after forced weaning [9], suggesting a decline in the milk production. The expression of the genes involved in milk production, such as whey acidic protein, β-casein, α-lactalbumin, and GLUT1, is acutely suppressed within a day of weaning [10, 11]. It has been also suggested that the ion transport systems via mammary epithelial cells are modulated after weaning. Tolkunov and Markov [12] have shown that the transepithelial potential difference in mouse mammary alveoli (approximately −18 mV when suckled, luminal side negative) falls toward zero between 5 and 20 h after forced weaning, while the transepithelial resistance was preserved. They have suggested a reduction in the ion concentration gradients (i.e., a decrease in the vectorial ion transport) between the interstitial fluid and milk after weaning. Thus, it has been assumed that weaning alters the function and/or expression of ion channels and transporters that contribute to the selective transepithelial ion transport. However, there is no direct evidence for the alteration of the ion channels in MS cells after weaning.

To address this issue, we examined the Kir currents in mouse MS cells before and after forced weaning using a whole-cell patch-clamp technique. We also analyzed the effect of forced weaning on the expression and localization of Kir2.1 in mammary glands. Our work provides the first evidence that the ion permeability of the MS cells is regulated during the weaning period, suggesting its involvement in the cessation of lactation.

## Materials and Methods

### Animals

The animal experimental procedures were carried out in accordance with Regulations on Management and Operation of Animal Experiments at the Obihiro University of Agriculture and Veterinary Medicine (OUAVM), and were approved by OUAVM Animal Care and Use Committee (approval No.24-64, 25–40, 25–41, 26–51, and 27–19). Female and male C57BL/6J mice obtained from Nihon SLC (Shizuoka, Japan) and their offsprings were used for the experiments. The mice were housed at 22°C with a 12 h:12 h light:dark cycle, with free access to drink and food.

### Forced weaning and teat sealing

After parturition, the dams were allowed to nurse their pups until mid-lactation to obtain the active mammary glands. In the experiment assessing the effect of forced weaning (“forced-weaning experiment”), the dams were separated from their pups at mid-lactation; their mammary glands were collected 1 day (24 h) after forced weaning. In the experiment evaluating the effect of milk stasis (“teat-sealing experiment”), the teats of unilateral abdominal and inguinal mammary glands of lactating mice were sealed with surgical adhesive (Aron Alpha A; Sankyo, Tokyo, Japan) at mid-lactation. Twenty-four hours after sealing, the sealed mammary glands and contralateral control mammary glands were collected for the analyses. Ages, durations of lactation, litter size, body weights of the dams, and wet weights of abdominal mammary glands in each experimental group are summarized in Tables 1 and 2.

**Table 1:** Data of mice in the forced-weaning experiment.

	Forced-weaning experiment							
	RT-PCR, histology		Immunohistochemistry		Western blotting		Patch-clamp	
	Lac	Post-W	Lac	Post-W	Lac	Post-W	Lac	Post-W
	N = 4	N = 4	N = 3	N = 3	N = 4	N = 4	N = 7	N = 5
Age (wks)	16.3 ± 1.5	15	17	17	17.8 ± 1.2	17.5 ± 1	19.6 ± 0.9	20.2 ± 1.9
	(13–15)				(16–20)	(16–19)	(17–23)	(16–26)
Lac Duration (days)	11	10	11.3 ± 1.1	10.7 ± 0.8	13.3 ± 0.6	13.0 ± 0.7	14.6 ± 1.0	13.8 ± 0.8
			(10–13)	(10–12)	(12–14)	(12–14)	(12–18)	(11–15)
Litter size	8.3 ± 0.3	6.3 ± 1.6	7.7 ± 1.1	7.3 ± 0.4	7.3 ± 0.7	7.0 ± 1.1	6.4 ± 0.6	6.2 ± 0.2
	(8–9)	(3–9)			(6–9)	(5–9)	(5–8)	(6–7)
Dam weight (g)	34.3 ± 0.7	34.1 ± 0.9	32.5 ± 2.0	32.6 ± 1.3	32.8 ± 0.8	30.9 ± 1.2	n.d.	n.d.
AM weight (mg)	438 ± 32	551 ± 79	n.d.	n.d.	383 ± 29	685 ± 58 *	n.d.	n.d.

Age, duration of lactation, litter size, body weight of dam and wet weight of abdominal mammary gland (AM) of the lactating (Lac) and post-weaning (Post-W) mice were summarized. Data are shown as mean ± SE. The numbers in parentheses represent minimal and maximal values. n.d., no data.

*, *p* < 0.05 *vs*. lactating.

**Table 2:** Data of mice in the teat-sealing experiment.

	Teat-sealing experiment			
	mRNA, histology		Patch-clamp	
	Control	Seal	Control	Seal
	N = 4		N = 5	
Age (wks)	16.8 ± 1.2		19.4 ± 3.5	
	(16–19)		(14–27)	
Lac Duration (days)	11		15.4 ± 1.4	
			(12–18)	
Litter size	8.0 ± 1.2		7.2 ± 0.5	
	(6–10)		(6–9)	
Dam weight (g)	34.4 ± 1.1		n.d.	
AM weight (mg)	412 ± 24	887 ± 55*	n.d.	n.d.

Age, duration of lactation, litter size, body weight of dam and wet weight of abdominal mammary gland (AM) were summarized. Data are shown as mean ± SE. The numbers in parentheses represent minimal and maximal values. n.d., no data.

*, *p* < 0.05 *vs*. control with paired *t*-test.

### Cell preparation for patch-clamp procedure

The MS cells were isolated from the mammary glands using digestive enzymes, as previously reported [8]. Briefly, the mice were euthanized by cervical dislocation, and the inguinal, abdominal, and/or thoracic mammary glands were collected. The mammary glands were minced and incubated in a divalent cation-free standard solution containing (in mM) 145 NaCl, 5 KCl, 10 HEPES, 10 glucose, and 4.6 NaOH at pH 7.4, supplemented with collagenase (type I, 300 U/ml; Wako, Osaka, Japan) and hyaluronidase (100 U/ml; Sigma-Aldrich, St. Louis, MO, USA), and gassed with 100% O_2_ for 30 min at 37°C. After gentle trituration with a pipette, the tissue was incubated once more for 30 min in the fresh digestion buffer. The cells filtered through 100 μm nylon mesh were washed with, and resuspended in, the divalent cation-free standard bath solution and stored at 4°C.

### Patch-clamp experiment

The cell preparations described above were used for the patch-clamp analyses within 6 hours of the isolation. The cells were plated out onto a non-coated borosilicate coverglass (Matsunami Glass Industry, Osaka, Japan) in a chamber mounted on an inverted microscope and were left for a few minutes to attach to the glass. Attached cells were initially superfused with the standard bath solution (in mM): 145 NaCl, 5 KCl, 10 HEPES, 10 glucose, 1 CaCl_2_, 1 MgCl_2_, and 4.6 NaOH at pH 7.4. Current recordings were made using an EPC7 amplifier (HEKA Electronik, Lambrecht, Germany) in the whole-cell configurations of the patch-clamp technique [13]. Patch-clamp pipettes (G-1.5; Narishige, Tokyo, Japan) have resistance of 5–10 MΩ when filled with the standard pipette solution (in mM): 110 K-glutamate, 10 KCl, 10 HEPES, 10 EGTA, 10 glucose, 1 MgCl_2_, and 25 KOH at pH7.4. To minimize the Na^+^ and Cl^−^ current, a NMDG-glutamate-rich bath solution containing (in mM) 145 NMDG-glutamate, 5 K-glutamate, 10 HEPES, 10 glucose, 1 CaCl_2_, 1 MgCl_2_, and 5.6 NMDG-OH at pH 7.4 was used. In some experiments, BaCl_2_ (dissolved in pure water to 1 M concentration) was diluted in the NMDG-glutamate-rich bath solution to the final concentration of 10^−4^ M. All experiments were performed at room temperature. Bath solutions were changed by gravity feed from reservoirs. The reference electrode (Ag–AgCl) was connected to the bathing solution via an agar bridge filled with the standard bath solution described above.

The whole-cell currents were filtered at 1 kHz with an internal four-pole Bessel filter and sampled at 2 kHz using the PowerLab system (AD Instruments, Sydney, Australia). For the whole-cell current measurements, the amplifier was driven by Scope software (AD Instruments) to allow the delivery of voltage-ramp protocol with the concomitant digitization of the current. Membrane potential was held at the holding potential of −45 mV. Currents were elicited and recorded using an 800-ms ramp pulse (commanded from −105 mV to +45 mV) with a 210-ms prepulse at −105 mV. The capacitive transient current was compensated. The average series resistance (*R*s), which was 18.2 ± 0.9 MΩ (n = 34), was not electrically compensated. In the figures showing the current–voltage (*I*-*V*) relationships, the voltages were not corrected for the voltage decrease across the *R*s. However, when slope conductances were estimated, the actual membrane voltages were determined by subtracting the dropped voltage from the commanded voltage. The pipette potential was corrected for the liquid junction potential between the pipette solution and the external solution, and between the external solution and the agar bridge, as described elsewhere [14].

### Analyses of mRNA expression and histological changes in mammary glands

Mice were euthanized by cervical dislocation. The collected abdominal mammary glands were stored in RNAlater (Life Technologies, Carlsbad, CA, USA) at −80°C for mRNA expression analyses. Parts of the inguinal mammary glands were fixed in 4% paraformaldehyde in 0.1 M phosphate buffer at 4°C for histological analyses.

The abdominal mammary glands were homogenized by vigorous agitation with zirconia beads (Micro Smash; TOMY, Tokyo, Japan) and total RNA was extracted from the homogenate with TRIzol (Life Technologies). The total RNA was purified on silica membrane columns (RNeasy; QIAGEN, Hilden, Germany) according to the manufacturer’s protocols. To obtain the cDNA, the purified total RNA (2 μg) was first incubated with 1 μg of oligo(dT) primer at 70°C for 5 min. It was reverse-transcribed using 200 units of Moloney murine-leukemia virus reverse transcriptase (Promega, Madison, WI, USA), 24 units of RNase inhibitor, and 12.5 nmol of dNTPs in a total volume of 25 μl at 42°C for 1 h, followed by heating at 94°C for 5 min. The genes of interest were amplified using a conventional PCR in 25 μl of the reaction solution containing *Taq* polymerase (0.625 U, Promega), MgCl_2_ (37.5 nmol), dNTPs (5 nmol), and sense and antisense primers (25 pmol each). Samples were subjected to 94°C for 2 min followed by 30 cycles of 30 s at 94°C, 30 s at 57°C, and 30 or 60 s at 72°C and finally incubated at 72°C for 5 min. PCR products were electrophoresed on 2% agarose gels containing ethidium bromide. To quantify the mRNAs for Kir2.1 and β-actin, their expression levels were measured using real-time PCR with respective cDNA fragments as standards (1 × 10^2^–10^8^ copies/μl). Real-time PCR was performed in a StepOne real-time PCR system (Life Technologies) using SYBR Green as a double-strand DNA-specific dye (KAPA SYBR FAST qPCR Kit; Nippon Genetics, Tokyo, Japan) according to the manufacturer’s protocol. Briefly, fluorescence was measured in every amplification cycle (3 s at 95°C and 30 s at 60°C) and the values for the threshold cycle (Ct) were determined for the standards and samples. The amount of a targeted gene in a sample was determined from the standard curve of Ct values against the amount of standard. Primers used were 5′-TCT TGG GAA TTC TCA CTT GC-3′ and 5′-AAC TCA GCT GAC ATC CAG AG-3′ for mouse Kir2.1, 5′-GGC AAC CTC ATG AAC CAG ATC-3′ and 5′-GCA AAG CAC ATT GCT GAG GAG G-3′ for mouse leukemia inhibitory factor (LIF), and 5′-CAG CTT CTT TGC AGC TCC TT-3′ and 5′-ATA GGA GTC CTT CTG ACC CA-3′ for mouse β-actin. The measurements were performed in duplicate.

The fixed mammary glands were embedded in paraffin, sliced into 5-μm thick sections, and stained with hematoxylin and eosin (HE). The area of the lumen, as an indicator of milk accumulation, was measured using Image J software (ver1.48; National Institute of Health, Bethesda, MD, USA). To minimize the observer bias, all the luminal structures (30–79 lumina) in 4 pictures, randomly selected from 50 pictures of a mammary gland, were analyzed, and the averaged value of the luminal areas was used for each mammary gland.

### Western blotting for Kir2.1

Dams of similar age, lactating duration, and litter size were paired. One mouse in each pair was assigned to the lactation group and the other to the post-weaning group. Preparation of protein lysate was performed simultaneously for each pair. MS cells were isolated from inguinal, abdominal, and thoracic mammary glands as described in the section entitled “Cell preparation for the patch-clamp procedure”. The isolated cells were resuspended in 0.25 M sucrose solution containing protease inhibitor cocktail (Complete; Roche, Manheim, Germany) and homogenized using Potter-Elvehjem grinder on ice. The homogenate was centrifuged at 9,000 × *g* for 10 min at 4°C and the supernatant was centrifuged at 100,000 × *g* for 90 min at 4°C. The precipitate (microsomal fraction) was solubilized in a membrane protein buffer (Cell-LyEx MP; TOYO B-Net, Tokyo, Japan). The protein concentrations of the microsomal fractions were determined using Lowry method with BSA as a standard. The proteins in the lysate (35 μg) were separated using 10% SDS-PAGE and transferred onto polyvinylidene difluoride (PVDF) membranes (Millipore, Bedford, MA, USA). The membranes were incubated first in blocking buffer [20 mM Tris/HCl (pH 7.4), 150 mM NaCl, containing 0.1% Tween 20 and 5% skimmed milk]. They were probed with anti-Kir2.1 antibody (1:2,000 dilution in the blocking buffer; ab85492, Abcam, Cambridge, UK) or anti-E-cadherin antibody (1:4,000 in the blocking buffer; #610181, Becton, Dickinson and Company, Franklin Lakes, NJ, USA) at 4°C overnight. The bound primary antibody was detected with horseradish peroxidase (HRP)-linked anti-mouse immunoglobulin (1:4,000 dilution in the blocking buffer; GE Healthcare, Little Chalfont, UK) and an enhanced chemiluminescence HRP substrate (Wako) on X-ray film. The densities of the bands were analyzed using the Image J software.

### Immunohistochemical staining for Kir2.1

Mice were deeply anesthetized with an intraperitoneal injection of pentobarbital (0.2 mg/g body weight) and sacrificed by cutting right atrium. Then, the 4% paraformaldehyde fixative was perfused from the left ventricle. The fixed abdominal mammary glands of lactating and post-weaning mice were collected, dehydrated in alcohol, embedded in paraffin, and sliced into 3-μm-thick sections. Immunohistochemistry for Kir2.1 in mammary gland paraffin sections was performed using rabbit anti-Kir2.1 antibody (ab65796; Abcam) as described in the previous study [15]. Briefly, for antigen retrieval, the sections were heated at 105°C for 15 min in 20 mM Tris-HCl buffer (pH 8.0) after deparaffinization. The sections were incubated with anti-Kir2.1 antibody (1: 800) or control rabbit IgG (Santa Cruz Biotechnology, Dallas, TX, USA) overnight at 4°C, and then incubated with biotin-conjugated anti-Rabbit IgG Abs and horseradish peroxidase-conjugated streptavidin (Nichirei, Tokyo, Japan). For signal development, 3,3′-diaminobenzidine (DAB) tetrahydrochloride-H_2_O_2_ solution was used. The sections were counterstained with Mayer’s hematoxylin.

### Statistics

Data are expressed as mean ± SE (standard error of the mean). Statistical analysis of the data was performed using a standard two-sample Student’s *t*-test or paired two-sample Student’s *t*-test, after confirming the equal variances of the two data sets with the F-test. *p* values less than 0.05 were considered statistically significant.

## Results

### Histological changes in mammary acini after forced weaning

**Fig 1:**
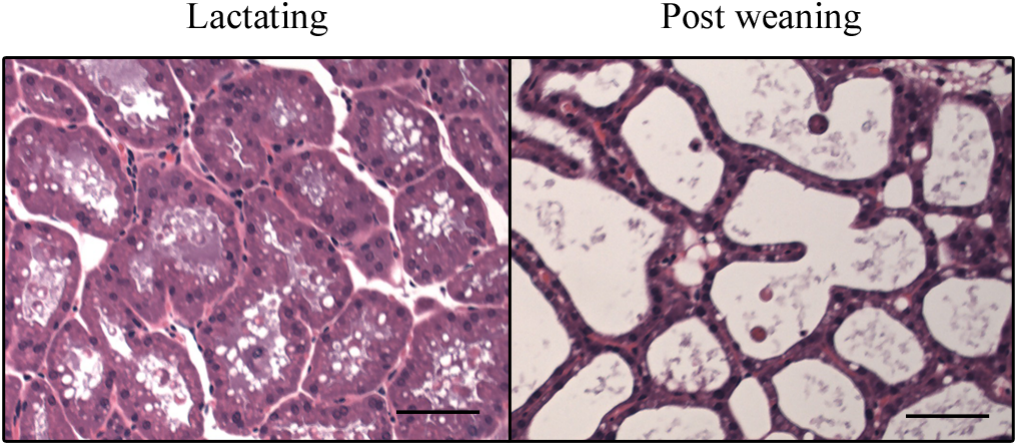
Structure of mammary acini in lactating and post-weaning mice. Sections of lactating and post-weaning mammary glands were stained with HE. The representative pictures are shown. The black bars indicate 50 μm.

To evaluate the morphological changes after weaning, the mammary glands collected a day (24 h) after forced weaning were compared with the glands at day 11 of lactation. Histological analysis showed that the lumina of acini in lactating mammary glands were lined with cuboidal secretory cells and in the post-weaning glands, by flattened secretory cells (Fig 1). The cross-section area of acinar lumen in the post-weaning glands was significantly greater than in lactating glands (1510 ± 182 μm^2^ and 3545 ± 387 μm^2^ for lactating and post-weaning gland, n = 4 and 3, respectively; *p* < 0.05). These results showed that the forced weaning induced the expansion of acini by milk accumulation.

### Effect of forced weaning on Kir current in MS cells

**Fig 2:**
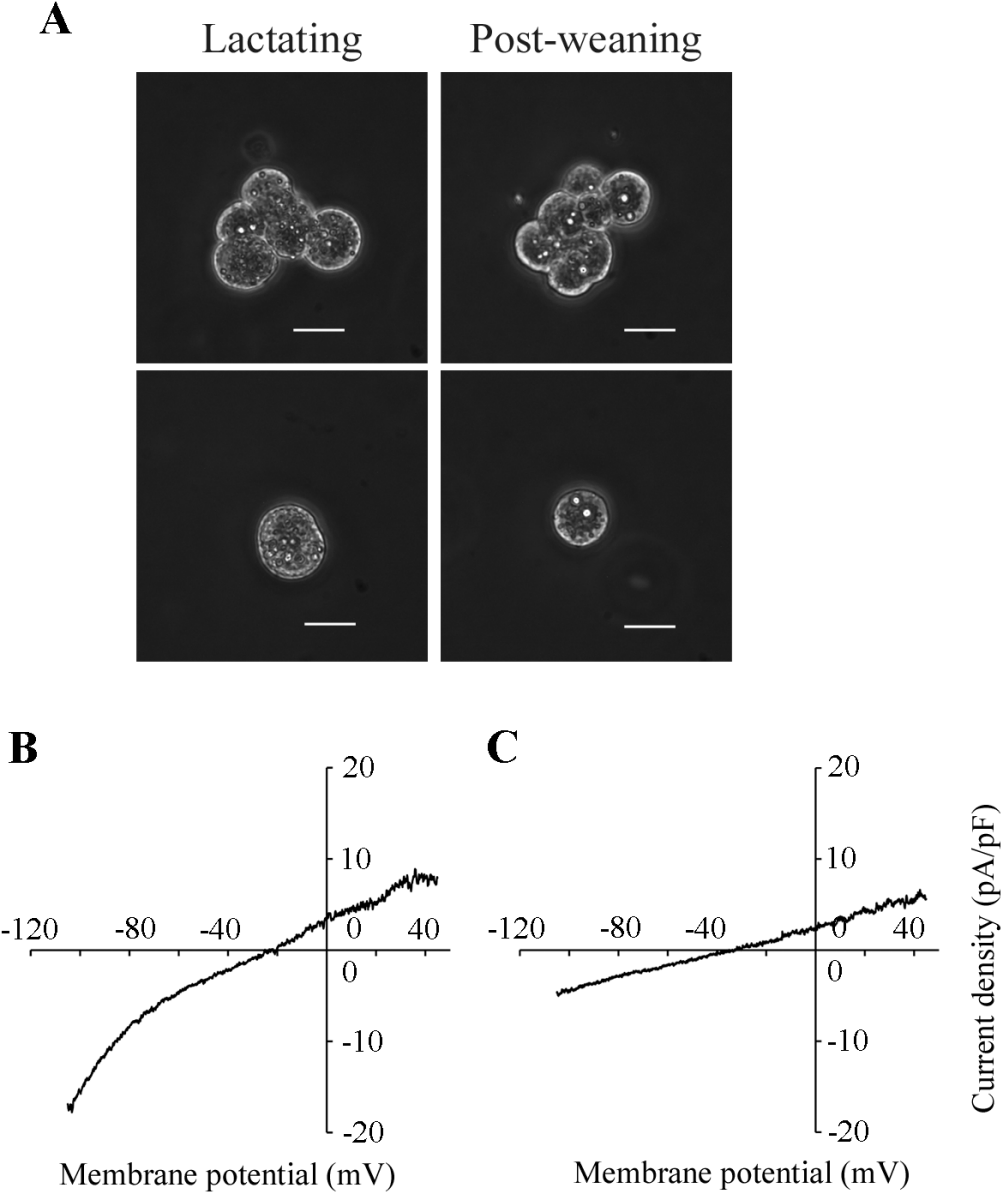
Whole-cell currents in MS cells of lactating and post-weaning mice. ***A*:** representative MS cells. Cell clusters (upper panels) and single cells (lower panels) obtained from lactating and post-weaning mammary glands are shown. Single cells were used for patch-clamp analyses. White bars indicate 20 μm. ***B*, *C*:** representative relationships between whole-cell currents and membrane potentials (*I*-*V*) in lactating and post-weaning MS cells. The lactating (*B*) and post-weaning (*C*) cells were perfused with the standard pipette solution (K-glutamate-rich solution) and the standard bath solution (NaCl-rich solution). The whole-cell currents were elicited by 800-ms ramp pulses from −105 to +45 mV.

**Fig 3:**
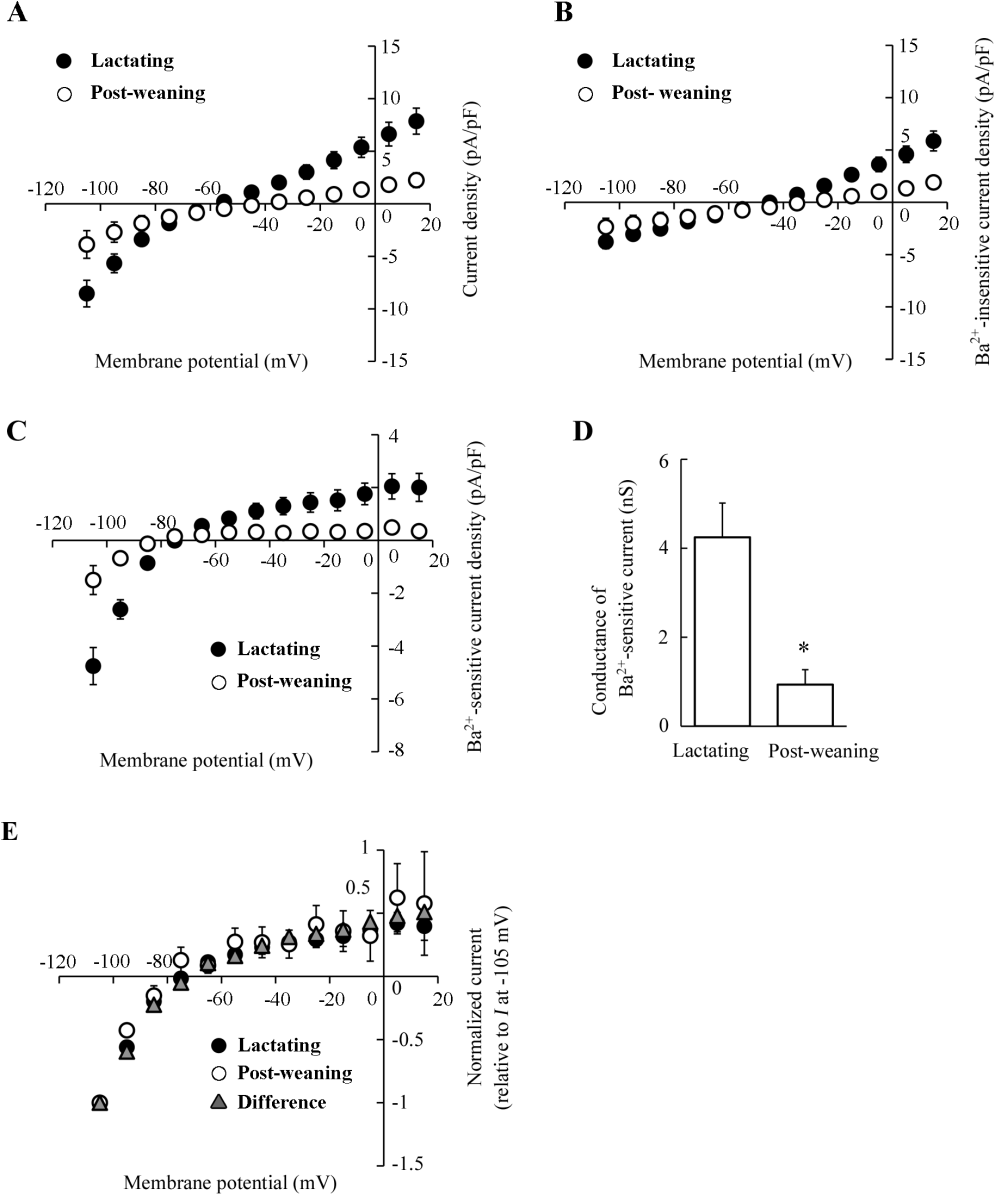
The effects of forced weaning on the Kir current in MS cells. ***A*-*C***: *I*-*V* relationships of the whole-cell currents in lactating and post-weaning MS cells. The control and Ba^2+^-insensitive currents were recorded in the NMDG-Cl-rich bath solution in the absence and presence of external Ba^2+^ (10^−4^ M), respectively. Ba^2+^-sensitive currents were determined by subtracting the Ba^2+^-insensitive currents from the control currents recorded in the same cells. The *I*-*V* relationships of control currents (*A*), Ba^2+^-insensitive currents (*B*), and Ba^2+^-sensitive currents (*C*) at the potentials from −105 to +15 mV with 10 mV intervals in lactating (filled circles) and post-weaning (open circles) MS cells are shown. Each point represents the mean ± SE (n = 10 and 11 for lactating and post-weaning, respectively). ***D***: slope conductances of Ba^2+^-sensitive inward currents of lactating and post-weaning MS cells. The slope conductances were determined from *I*-*V* relationships at the potentials between −105 and −85 mV. Data represent the mean ± SE (n = 10 and 11 for lactating and post-weaning, respectively). *, *p* < 0.05 vs. lactating cells. ***E***: *I*-*V* relationships of normalized currents. The currents in lactating (filled circles) and post-weaning (open circles) MS cells were normalized to the current at −105 mV. The differential current (triangles) between averaged Ba^2+^-sensitive currents of lactating and post-weaning cells was also normalized.

The changes in the ion permeabilities in MS cells were examined using a whole-cell patch-clamp technique. The MS cells were isolated from mammary glands of mice at mid-lactation or 24 h after forced weaning. The majority of the isolated MS cells were round, in both lactating and post-weaning mammary glands. The diameter of post-weaning MS cells was slightly smaller than the diameter of lactating MS cells, as shown in Fig 2A. In the whole-cell configuration, the cell capacitances of MS cells in the post-weaning mice (16.0 ± 1.7 pF, n = 11) were also slightly smaller than in lactating mice (19.2 ± 1.3 pF, n = 10), although the difference was not significant. We have reported that MS cells of lactating mice have the Kir conductance with electrophysiological characteristics similar to those of Kir2.1 [8]. The native Kir current in the MS cells is highly sensitive to external Ba^2+^ (*K*_d_ = 5.1 μM at −105 mV) [8]. When lactating MS cells were perfused with K-glutamate-rich pipette solution and NaCl-rich bath solution, the whole-cell currents displayed the inward rectification at the potentials more negative than the equilibrium potential of K^+^ (*E*_K_ ≈ −86 mV) (Fig 2B). However, MS cells of post-weaning mice showed little inward rectification (Fig 2C), suggesting that the whole-cell current, including Kir current in MS cells, was reduced by forced weaning. To demonstrate the alteration of Kir current amplitude clearly, Ba^2+^ (10^−4^ M)-sensitive Kir current in MS cell was determined under the NMDG-glutamate-rich conditions (Na^+^ and Cl^−^ in the bath solution were mostly replaced by NMDG and glutamate, respectively). The Ba^2+^-sensitive Kir current was calculated by subtracting the current after the Ba^2+^ treatment (Ba^2+^-insensitive current) from the current before the treatment (control current). The Fig 3A, 3B and 3C show the *I*-*V* relationships for control, Ba^2+^-insensitive, and Ba^2+^-sensitive whole-cell currents, respectively. As shown in Fig 3A, MS cells isolated from lactating mice exhibited greater inwardly rectifying current than those from mice after weaning. The Ba^2+^-sensitive current in lactating MS cells exhibited inwardly rectifying currents, whose reversal potentials (~ −81 mV) were close to the value of *E*_K_ (Fig 3C), confirming the successful isolation of Kir current. The Ba^2+^-sensitive Kir current was detected also in the post-weaning MS cells. As shown in Fig 3E, the *I*-*V* relationship of normalized current was almost the same in lactating and post-weaning cells. However, the current amplitude in post-weaning MS cells was lesser than in lactating MS cells (Fig 3C). The averaged slope conductance of the Ba^2+^-sensitive inward currents in the post-weaning MS cells (0.93 ± 0.34 nS) was significantly lower than in the lactating MS cells (4.25 ± 0.77 nS; Fig 3D). Moreover, the plot of the differences between the Ba^2+^-sensitive currents in lactating cells and post-weaning cells (representing the inhibited current) had the same shape as the Kir-current plot for the lactating MS cells (Fig 3E). These results indicated that the Kir conductance in lactating MS cells decreased a day after weaning.

### Effect of forced weaning on the expression of Kir2.1 mRNA and protein

**Fig 4:**
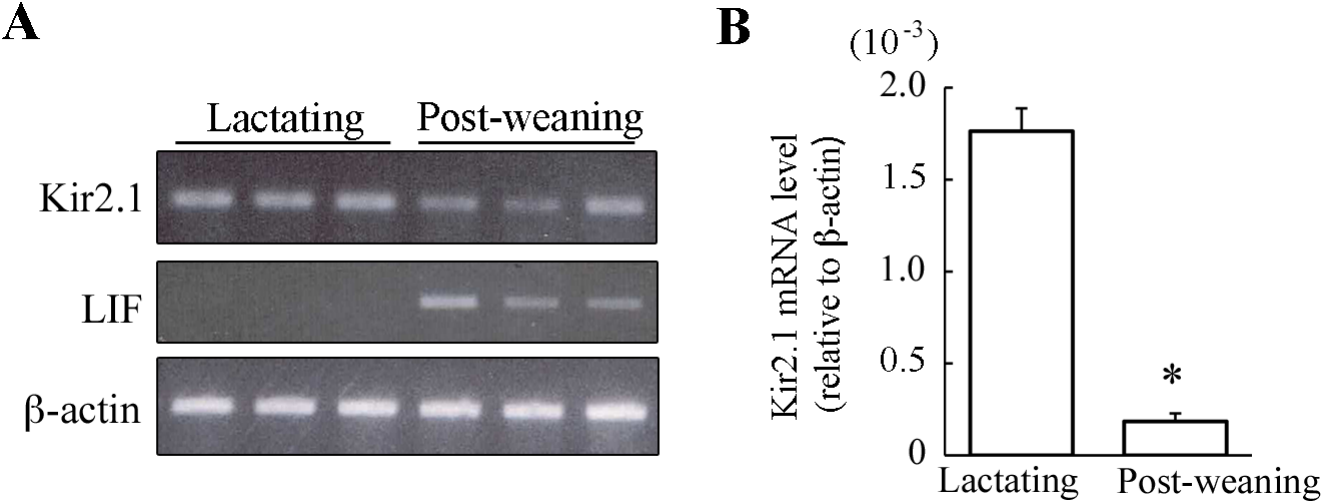
The effects of forced weaning on the mRNA expression of Kir2.1 in the mammary gland. ***A***: mRNA expressions for Kir2.1, LIF, and β-actin in mammary glands. Total RNA was extracted from the abdominal mammary glands of lactating mice and 24-h post-weaning mice. The mRNA expression was examined using conventional RT-PCR. The representative amplifications obtained from three different mice are shown. ***B***: Quantitative analysis of Kir2.1 mRNA expression. The expression levels of Kir2.1 mRNA (relative to β-actin) in lactating and post-weaning mammary glands were examined using real-time PCR. Data represent the mean ± SE (n = 4 for each). *, *p* < 0.05 vs. lactating glands.

It has been strongly suggested that Kir2.1 channel at least partly contributes to the Kir current in the lactating mouse MS cells [8]. Therefore, the mRNA expression of Kir2.1 in mammary glands obtained from mice at day 11 of lactation and 24 h after separation from their pups at day 11 of lactation was analyzed. LIF, one of the involution inducers [16, 17], was also investigated to confirm the progression of the involution process. In agreement with the previous reports, the LIF mRNA was clearly detected in the mammary gland of post-weaning mice, but not in lactating mammary glands. Although Kir2.1 mRNA was detected in both types of mammary glands, the bands of Kir2.1 from post-weaning glands were fainter than the bands from lactating mammary glands (Fig 4A). The real-time PCR analyses demonstrated that the level of Kir2.1 mRNA expression in the mammary gland of post-weaning mice was 90% lower than in the lactating mammary gland (Fig 4B). Also in mice at day 15 of lactation, forced weaning reduced the Kir2.1 mRNA expression by 97% (n = 4 and 3 for lactating and post-weaning mice, data not shown). These results showed that forced weaning at mid-lactation lowered the abundance of Kir2.1 transcripts. Thus, we could speculate that the reduction in the levels of Kir2.1 transcripts after weaning had caused the downregulation of the Kir conductance by reducing the *de novo* synthesis of Kir2.1 protein.

**Fig 5:**
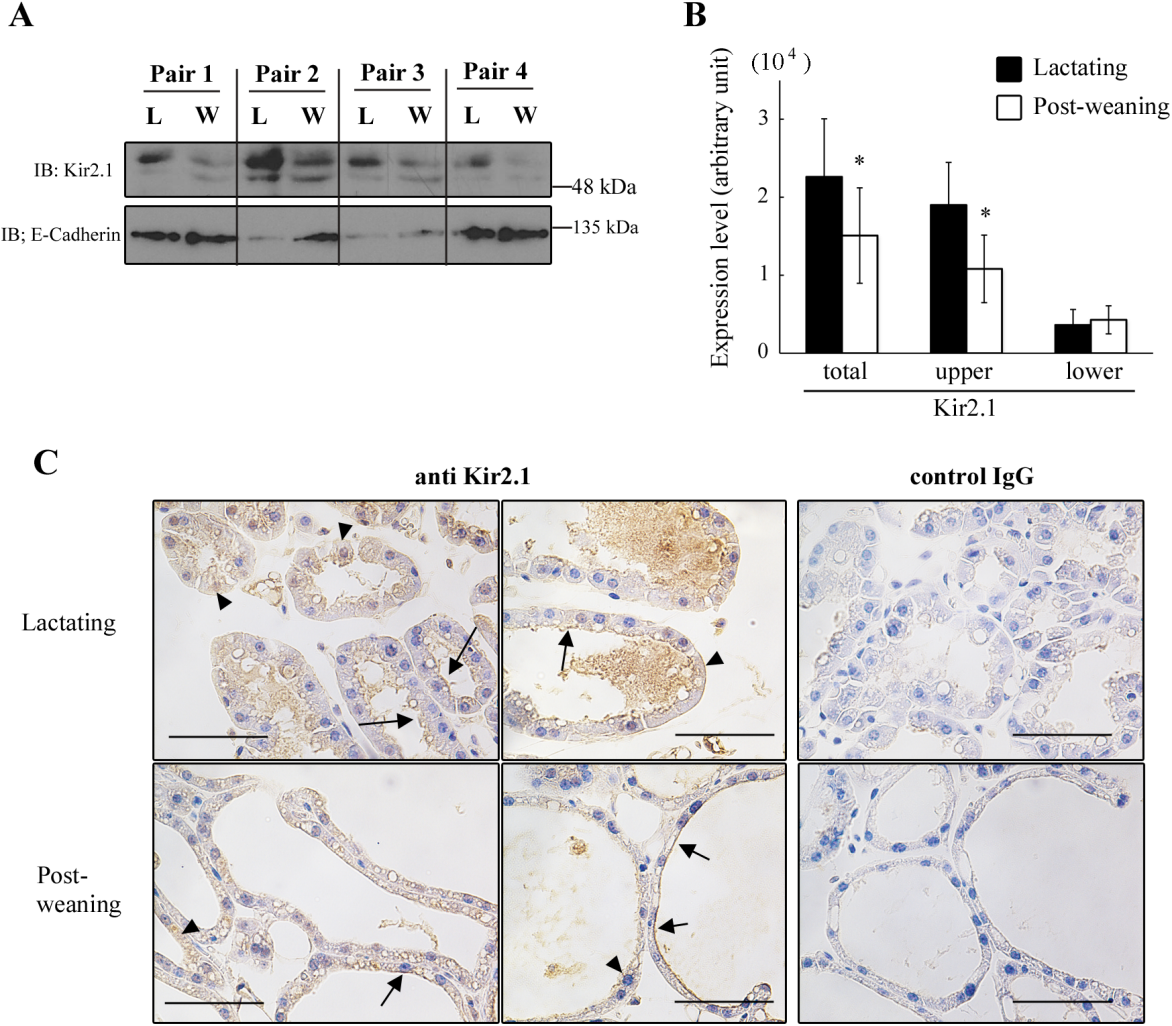
The effects of forced weaning on the protein expression of Kir2.1 in MS cells. ***A*, *B***: expression of Kir2.1 protein in the microsomal fraction of MS cells. Dams of a similar age, nursing duration, and litter size were paired. The cell isolation and enrichment of microsomal fraction were performed simultaneously for each pair. The lysate (35 μg of protein) of the microsomal fraction from lactating (L) and post-weaning (W) MS cells were separated with SDS-PAGE and immunoblotted with the anti-Kir2.1 and anti-E-cadherin antibody (*A*). Two bands around 50 kDa are shown. The densities of the bands were measured (*B*). Data represent mean ± SE (n = 4 for each). *, *p* < 0.05 vs. lactating, using paired *t*-test. ***C***: localization of Kir2.1 in mammary glands. Sections of lactating (upper panels) and post-weaning (lower panels) mammary glands were immunostained with anti-Kir2.1 antibody and control IgG (for negative control). Arrows indicate cells stained at the apical membrane. Arrowheads indicate cells with diffuse staining in the cytoplasmic region and at the basolateral side of the cells. Black bars show 50 μm.

We next examined the effect of weaning on the expression of Kir2.1 protein in the microsomal fraction of MS cells. In Western blot analysis, anti-Kir2.1 antibody-reactive bands were observed in the microsomal fraction of the MS cells, with the size of approximately 50 kDa (Fig 5A). We have previously confirmed that the treatment of the microsomal lysate with N-glycosidase F shifts both bands to the predicted size of Kir2.1 (48 kDa) [8]. Therefore, a great proportion of Kir2.1 is in the N-glycosylated form in both lactating and post-weaning MS cells. The intensity of the upper band (highly glycosylated Kir2.1), which was abundant in lactating MS cells, decreased significantly in the post-weaning MS cells (Fig 5A and 5B). The relative expression levels of highly glycosylated, slightly glycosylated, and total Kir2.1 in post-weaning MS cells were 0.53 ± 0.08, 1.33 ± 0.30, and 0.63 ± 0.09, respectively, in comparison with their paired lactating MS cells. There was no significant difference between E-cadherin abundance in lactating and post-weaning mammary glands (Fig 5A). Thus, the forced weaning decreased the levels of Kir2.1 protein, especially in highly glycosylated form, in the microsomal fraction of MS cells.

We also investigated the localization of Kir2.1 in the mammary gland during lactation and after forced weaning. In our previous study, we have demonstrated a diffused staining of anti-Kir2.1 antibody in MS cells in lactating mammary glands [8]. Here, we used an improved, more sensitive immunohistochemical protocol to visualize the antigen localization. In the lactating mammary glands, positive staining of anti-Kir2.1 antibody was detected at the apical membrane of most MS cells (Fig 5C). However, a small fraction of the cells showed positive staining at the basolateral side and/or in the cytoplasmic region of MS cells rather than in the apical plasma membrane (Fig 5C). This may suggest that the localization of Kir2.1 was heterogeneously regulated at the cellular level. In mammary glands of post-weaning mice, the positive staining was detected at the apical membrane and/or in the cytosolic regions of the MS cells. The Kir2.1 at the plasma membrane of the post-weaning cells may contribute to the residual Kir whole-cell current shown in Fig 3C. There were no clear differences between the localization patterns in lactating and post-weaning mammary gland.

### Effect of teat sealing on Kir current in MS cells

**Fig 6:**
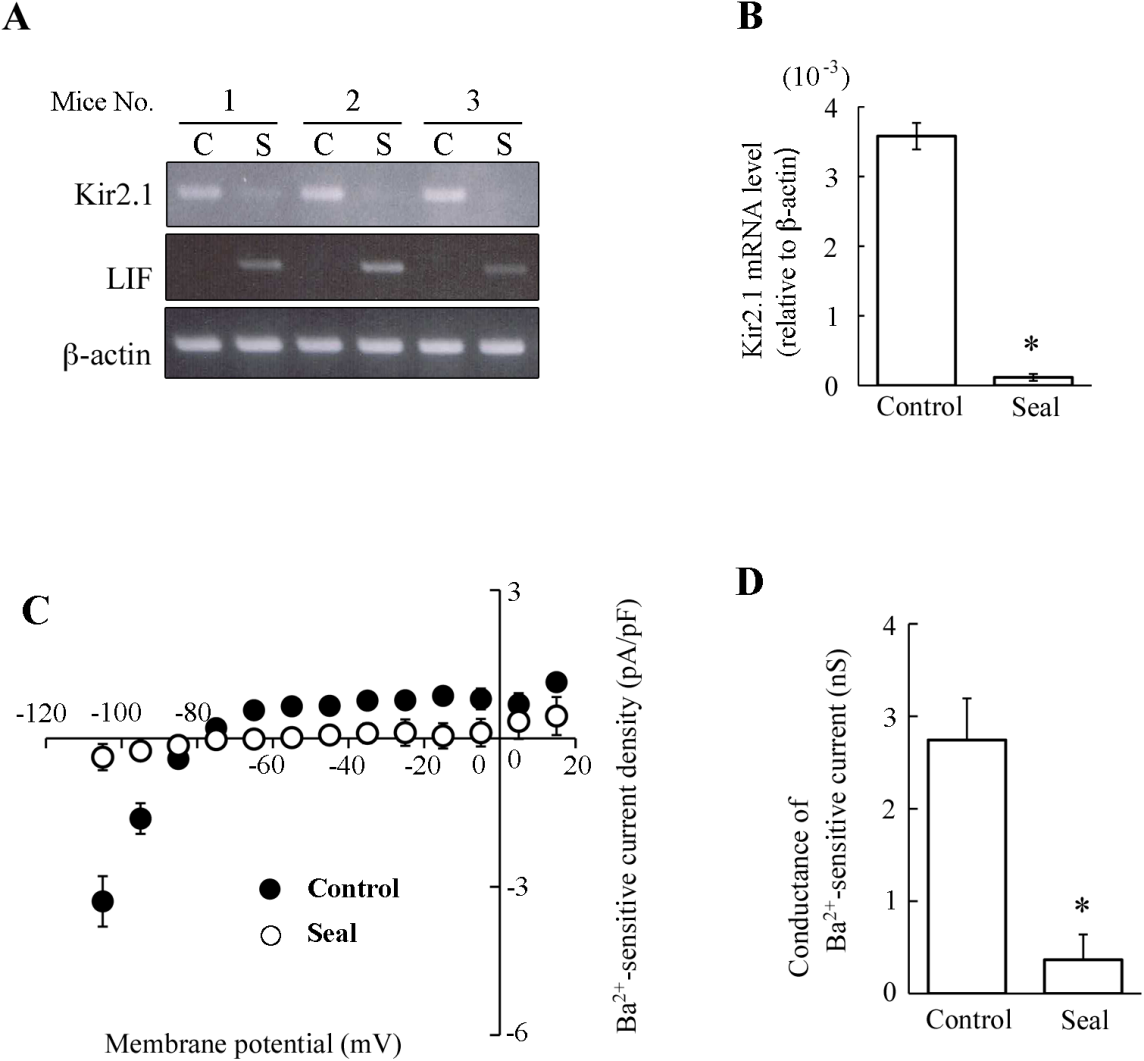
The effects of teat sealing on Kir2.1 mRNA expression in mammary glands and the Kir currents in MS cells. ***A***: mRNA expression of Kir2.1, LIF, and β-actin in mammary glands. At mid-lactation, the teats of unilateral mammary glands were sealed for 24 hours. Total RNA was extracted from the unsealed (control, C) and sealed (S) abdominal mammary glands. The mRNA expression was examined using the conventional RT-PCR. The amplification representative for three different mice is shown. ***B***: Quantitative analysis of Kir2.1 mRNA expression. The expression levels of Kir2.1 mRNA (relative to β-actin) in control and sealed mammary glands were examined using the real-time PCR. Data represent the mean ± SE (n = 4 for each). *, *p* < 0.05 vs. control with paired *t*-test. ***C***: *I*-*V* relationships of Ba^2+^-sensitive currents in MS cells of control and sealed mammary glands. The Ba^2+^-sensitive currents of control (filled circles) and sealed (open circles) MS cells were determined as described in the legend of Fig 3. Each point represents the mean ± SE (n = 5 and 8 for control and sealed glands, respectively). ***D***: Slope conductances of inward currents of MS cells from control and sealed mammary glands. The slope conductances were determined from *I*-*V* relationships at the potentials between −105 and −85 mV. Data represent the mean ± SE (n = 5 and 8 for control and sealed gland, respectively). *, *p* < 0.05 vs. control.

The forced weaning partly decreased the Kir conductance by reducing the Kir2.1 transcript levels. To clarify the mechanism of this regulation, the effects of teat sealing were examined. Sealed mammary glands accumulate milk in their lumen and start to involute locally while unsealed glands remain intact [17, 18]. Thus, by comparing the sealed (involuting) mammary gland and unsealed (lactating) mammary gland in the same mouse, i.e., under the same hormonal conditions, we can evaluate the local effects of milk stasis at the early phase of involution. We found that 24-h teat sealing increased the average lumen size of mammary acini in the sliced sections in comparison with unsealed control acini (1668 ± 268 and 3052 ± 554 μm^2^ for control and sealed mammary glands, respectively; *p* < 0.05). Moreover, the abundant expression of LIF mRNA in the sealed glands confirmed the onset of local involution (Fig 6A). Teat sealing, like forced weaning, reduced the expression of Kir2.1 mRNA by 97% (Fig 6A and 6B).

To investigate the effect of teat sealing on Kir conductance in MS cells, the cells were isolated from sealed and unsealed (control) glands, and were used in the whole-cell patch-clamp analyses. In the whole-cell configuration, the cell capacitances of MS cells isolated from the control and sealed mammary glands were 18.0 ± 3.0 pF (n = 5) and 17.8 ± 2.9 pF (n = 8), respectively. The control MS cells exhibited the Ba^2+^ (10^−4^ M)-sensitive Kir current (Fig 6C). However, the Kir current in the MS cells of the sealed mammary glands was almost abolished (Fig 6C). The averaged slope conductances of Ba^2+^-sensitive inward currents in the MS cells of the control and sealed mammary glands were 2.74 ± 0.45 nS and 0.36 ± 0.27 nS, respectively (Fig 6D). These results showed that teat sealing significantly depressed both Kir conductance and Kir2.1 mRNA expression. Thus, milk stasis may be sufficient to decrease the Kir conductance in MS cells. In contrast, the systemic hormones could not maintain Kir conductance.

## Discussion

The cellular functions involved in milk production are rapidly downregulated after weaning to stop the lactation [10, 11]. However, the weaning-induced alterations in MS-cell ion permeability have not been investigated so far. In the present study, we demonstrated that the Kir conductance, one of the ion permeabilities in lactating MS cells, decreased immediately after forced weaning in mice.

Mouse MS cells display Kir current, and Kir2.1 contributes to this current [8]. Taking advantage of the characteristics of the native Kir2.1-like channel, which is highly sensitive to external Ba^2+^ [8, 19], we recorded and compared the Ba^2+^ (10^−4^ M)-sensitive Kir conductances in lactating and post-weaning MS cells. In our previous work, we have characterized the Kir current in MS cells perfused with a NaCl-rich bath solution (containing 5 mM KCl). Here we examined the whole-cell current in an NMDG-glutamate-rich bath solution (containing 5 mM K-glutamate). The inward conductance and the degree of inward rectification of Ba^2+^-sensitive current measured in NMDG-glutamate-rich solution were similar to those measured in NaCl-rich bath solution (Fig 3C and 3D; [8]). Thus, it is plausible that the Ba^2+^-sensitive inwardly rectifying current in NMDG-glutamate-rich solution involves the same components as the Kir2.1-like current measured in NaCl-rich solution.

The Ba^2+^-sensitive Kir2.1-like current in post-weaning MS cells was lower than that in lactating MS cells (Figs 2B, 2C, 3C and 3D). The suppressed current, i.e., the difference between the Ba^2+^-sensitive current in lactating and post-weaning cells, displayed the *I*-*V* relationship identical to the Kir2.1-like current in lactating MS cells (Fig 3E). Thus, we suggest that Kir2.1-like Kir current in lactating MS cells decreases immediately after weaning. This decrease in the Kir conductance in post-weaning MS cells was accompanied by a decrease in the expression of Kir2.1 mRNA (Fig 4A and 4B) and Kir2.1 protein, especially in its highly glycosylated form, in the microsomal fraction (including plasma membrane and membrane of intracellular organelle like ER) (Fig 5A and 5B). It is possible that weaning decreases the Kir2.1 mRNA level by modulating its transcriptional activity and/or its degradation, because previous reports have shown that the weaning activates and deactivates many transcriptional factors [20–23] and shortens the half-life of casein mRNA [24]. The reduction in the levels of the Kir2.1 mRNA would decrease *de novo* synthesis of Kir2.1 protein, reducing the amounts of mature Kir2.1 protein at the cell surface and lowering the Kir conductance.

However, the altered levels of Kir2.1 protein in the microsomal fraction (47% decrease) cannot completely account for the reduction in the Kir conductance (78%). This discrepancy suggests the existence of other regulatory mechanisms. Firstly, Kir2.1 may be deactivated in the post-weaning MS cells. It is known that the intrinsic membrane lipids like PIP_2_ and cholesterol modulate the activity of heterologously expressed Kir2.1 [19, 25, 26]. Such mechanisms may contribute to the reduction of the Kir conductance in the post-weaning MS cells. Secondly, the amount of Kir2.1 protein at the cell surface of post-weaning cells may be profoundly reduced, beyond the degree of alteration observed in the microsomal fraction. In our study, we could not quantitatively show the amount of Kir2.1 at the cell surface of the MS cells. It would be interesting to analyze these changes, focusing on the alterations in Kir2.1-specific trafficking systems [27, 28] and/or global apical membrane removal associated with the apocrine secretion and phagocytic uptake of milk fat globules [29, 30] during the post-weaning period. Further studies will be required to completely understand the mechanisms by which Kir conductance is reduced after weaning.

In consistence with the results from forced weaning experiments, milk stasis caused by teat sealing also suppressed Kir conductance and Kir2.1 mRNA expression in MS cells (Fig 6). After some of the teats of the dams had been sealed, their pups continued to increase their body weight (data not shown). This observation indicated that the systemic lactogenic hormone levels of dams was elevated to support lactation in the unsealed glands. Thus, our data suggest that milk stasis in the mammary acinar lumen (Fig 1) decreases the expression of Kir2.1 and the Kir conductance in MS cells at the early phase of weaning. The systemic hormones were not sufficient to maintain the original Kir conductance.

It is worth discussing the physiological significance of the weaning-related decrease of Kir conductance in MS cells. It has been considered that the Kir2.1-like potassium channel might contribute to the maintenance of the resting membrane potential and provide a driving force for the ions secretion. Therefore, the decrease of Kir conductance after weaning might cause the decrease in the driving force for vectorial ion (and fluid) transport across the secretory cell layer. Like the Ba^2+^-sensitive Kir conductance, Ba^2+^-insensitive conductance was also decreased in the post-weaning MS cells (Fig 3B). Although the Ba^2+^-insensitive current has not been completely characterized, the alterations in multiple ion channels and transporters may contribute to the changes in the transepithelial ion and fluid flux after weaning.

In conclusion, we demonstrated that forced weaning and milk stasis in the mammary acinar lumina immediately decrease the Kir conductance in MS cells. Our results strongly suggest that this decrease was partly caused by the reduction in the Kir2.1 mRNA and protein expression. These alterations at post-weaning period may be involved in the pausing of lactation. Further investigation of the ion permeabilities in MS cells and their post-weaning regulation should provide important clues to the nature and the mechanism of milk production and its cessation.
